# Postmortem submersion interval estimation of cadavers recovered from freshwater based on gut microbial community succession

**DOI:** 10.3389/fmicb.2022.988297

**Published:** 2022-12-02

**Authors:** Fuyuan Zhang, Pengfei Wang, Kuo Zeng, Huiya Yuan, Ziwei Wang, Xinjie Li, Haomiao Yuan, Shukui Du, Dawei Guan, Linlin Wang, Rui Zhao

**Affiliations:** ^1^Department of Forensic Pathology, China Medical University School of Forensic Medicine, Shenyang, China; ^2^Liaoning Province Key Laboratory of Forensic Bio-evidence Science, Shenyang, China; ^3^Institute of Evidence Law and Forensic Science, China University of Political Science and Law, Beijing, China

**Keywords:** forensic medicine, postmortem submersion interval, microbial community, aquatic habitats, decomposition

## Abstract

Microbial community succession during decomposition has been proven to be a useful tool for postmortem interval (PMI) estimation. Numerous studies have shown that the intestinal microbial community presented chronological changes after death and was stable in terrestrial corpses with different causes of death. However, the postmortem pattern of intestinal microbial community succession in cadavers retrieved from water remains unclear. For immersed corpses, the postmortem submersion interval (PMSI) is a useful indicator of PMI. To provide reliable estimates of PMSI in forensic investigations, we investigated the gut microbial community succession of corpses submersed in freshwater and explored its potential application in forensic investigation. In this study, the intestinal microbial community of mouse submersed in freshwater that died of drowning or CO_2_ asphyxia (i.e., postmortem submersion) were characterized by 16S rDNA amplification and high-throughput sequencing, followed by bioinformatic analyses. The results demonstrated that the chronological changes in intestinal bacterial communities were not different between the drowning and postmortem submersion groups. α-diversity decreased significantly within 14 days of decomposition in both groups, and the β-diversity bacterial community structure ordinated chronologically, inferring the functional pathway and phenotype. To estimate PMSI, a regression model was established by random forest (RF) algorithm based on the succession of postmortem microbiota. Furthermore, 15 genera, including *Proteus*, *Enterococcus*, and others, were selected as candidate biomarkers to set up a concise predicted model, which provided a prediction of PMSI [MAE (± SE) = 0.818 (± 0.165) d]. Overall, our present study provides evidence that intestinal microbial community succession would be a valuable marker to estimate the PMSI of corpses submerged in an aquatic habitat.

## Introduction

Postmortem interval (PMI) provides crucial information when conducting a medicolegal death investigation (Ashe et al., 2021), such as determination or exclusion of possible suspects in cases ruled a homicide. However, PMI estimation has been a challenging task in forensic practice. Many studies have been conducted and numerous methods developed to provide reliable PMI estimates. For example, changes in body temperature (algor mortis) (Brown and Marshall, 1974) and the temporary stiffening of the muscles (rigor mortis) (Bate-Smith and Bendall, 1949) might be useful for early PMI (within 48 h of death) prediction. Insect succession could be used to estimate longer PMI by identifying characteristic life stages that take well-defined amounts of time to develop (Matuszewski and Madra-Bielewicz, 2016). However, these methods were developed mainly based on experiences from the examination of corpses and terrestrial ecosystems. When the corpses were recovered from aquatic systems, including rivers, lakes, and seas (Heaton et al., 2010; Cartozzo et al., 2021b), they were not applicable due to the inherent difference on oxygen content, salinity, and necrophagous insects (Fenoglio et al., 2014; Wallace and Merritt, 2019; Benbow et al., 2020). Postmortem submersion interval (PMSI), the period between entry into the water and recovery of the dead body, is a good indicator of PMI for submerged corpses (Humphreys et al., 2013). Currently, visual assessment of the stages of decomposition (such as the total aquatic decomposition scores) is still the primary method of estimating the PMSI (van Daalen et al., 2017; Palazzo et al., 2020). Due to the susceptibility to subjective judgment and environment, these empirical methods would hardly provide accurate information in a medicolegal context (Christensen, 2004). Therefore, novel methods for PMSI estimation are desired in forensic practice.

Decomposition of vertebrate corpse is a multifactorial process, which is mediated by inhabited microbes internally and externally throughout the dead biomass (Javan et al., 2016a,b). In the last decade, multiple high-throughput omics technologies, including transcriptomics (Haas et al., 2021), proteomics (Choi et al., 2019), and metabolomics (Aiello et al., 2021), have been utilized to trace the chronological changes of various biomolecules for PMI estimation. Moreover, our recent study detected the dynamic changes of metabolites in the blood of rat corpses submerged in freshwater through non-targeted metabolomics and provided a novel random forest (RF) algorithm-based PMSI estimation model (Zhang et al., 2022). On the other hand, advances in sequencing platforms and computational pipelines have made it possible to study highly speciose microbial communities at an unprecedented depth, promoting the applications of microbiomics in PMI estimation (Metcalf et al., 2017). Metcalf et al. (2013) find postmortem microbial community changes are dramatic, measurable, and repeatable. This discovery has spurred vast amounts of research performed on human or animal remains decaying under multienvironment to study microbial community succession after death, which have been recognized as a potential approach to predict PMI or PMSI (Pechal et al., 2014; Benbow et al., 2015; Hyde et al., 2015; Wang et al., 2022). However, when it comes to corpses recovered from water, these microbiota studies are limited to the bacterial community associated with the submerged bones or colonized on the external surfaces of corpses (Dickson et al., 2011; Cartozzo et al., 2021b). Due to several factors (such as flora, fauna, microorganisms, water flow, and environmental parameters) that influence the process of decomposition, the external colonized microbial populations on water submersed corpses are easy to change (Benbow et al., 2015). It has been reported that the succession of gut microbial communities is relatively stable and not affected significantly by the cause of death in terrestrial ecosystems (Hauther et al., 2015), because the internal postmortem microbial communities are not susceptible to either abiotic (e.g., humidity, temperature, and pH) or biotic variables (e.g., gasses, insects, and scavenger activities) (Hauther et al., 2015; Oliveira and Amorim, 2018). Considering the complexity of aquatic systems, we hypothesized that the intestinal microbial community could be used to estimate the PMSI of corpses recovered from water.

To confirm this hypothesis, we conducted this study to monitor the mouse intestinal flora succession by 16S rDNA sequencing within the 14-day postmortem period in a natural aquatic environment in order to build up a feasible regression model for PMSI estimation.

## Materials and methods

### Study design and sample collection

The experiments were carried out in October in a natural fresh river (Shenyang, China; N41°57’, E123°27′). A total of 180 mice (strain C57BL/6J, 20–25 g, males, 8–10 weeks) were used and divided randomly into two parts. Intestinal content samples were obtained on drowning and postmortem submersion mice at the following time points: 0, 6, and 12 h, 1, 3, 5, 7, 10, and 14 d postmortem (designated as 0, 0.25, 0.5, 1, 3, 5, 7, 10, and 14 d, respectively). In the first part, 144 mice were separated into drowning (D, *n* = 72, 8 mice at each indicated time point) and postmortem submersion (PS, *n* = 72, 8 mice at each time point) subgroups as an exploratory experiment. In the drowning subgroup, animals were placed in sterile string bags and submerged repeatedly in cycles of 1 min submersion and 30 s withdrawing. After death, the mice were submerged in the natural fresh water. In the postmortem submersion subgroup, mice were sacrificed by CO_2_ suffocation and submerged in water under the same conditions as the drowning group (Wang et al., 2020; Zhang et al., 2022). For validation, part two was set up independently. Thirty-six mice were processed according to the steps described above (drowning subgroup: 18 mice; postmortem submersion subgroup: 18 mice; 2 mice at each time point). A summary of the animal groupings is shown in Table 1. All animal experiments were approved by the Animal Experiment Committee of China Medical University (Approval no: CMU2021202).

**TABLE 1:** Experimental design and animal groups.

Postmortem submersion interval	Part 1: Exploratory experiment (*n*)		Part 2: Validation experiment (*n*)	
	Drowning	Postmortem submersion	Drowning	Postmortem submersion
0 d	8	8	2	2
0.25 d	8	8	2	2
0.5 d	8	8	2	2
1 d	8	8	2	2
3 d	8	8	2	2
5 d	8	8	2	2
7 d	8	8	2	2
10 d	8	8	2	2
14 d	8	8	2	2

For bacterial genome deoxyribonucleic acid (DNA) extraction, cecal contents were collected into sterile 1.5 mL Eppendorf (EP) tubes in a sterile laboratory environment according to Chen et al. (2017). Diurnal temperatures ranged between 5^°^C and 15^°^C during sampling. All samples were immediately frozen in liquid nitrogen and stored at −80^°^C for subsequent sequencing. Prior to the animal experiments, 5 water samples (1 L each) were filtered through sterile 0.2 μm membrane filters (Fisher Scientific, Hampton, NH) to investigate microorganisms from the environment. These samples were also stored at −80^°^C for further analysis.

### Deoxyribonucleic acid extraction, polymerase chain reaction amplification, and sequencing

Total genome DNA from samples was extracted using the CTAB method. DNA concentration and purity was monitored on 1% agarose gels. According to the concentration, DNA was diluted to 1 ng/μL using sterile water. 16S rDNA region V3–V4 was amplified with a specific combination of primers (341F-5′-CCTACGGGNGGCWGCAG-3′ and 806R-5′-GGACTACHVGGGTATCTAAT-3′). All Polymerase chain reaction (PCR) reactions were carried out with 15 μL of Phusion High-Fidelity PCR Master Mix (New England Biolabs), 2 μM of forward and reverse primers, and approximately 10 ng of template DNA. Thermal cycling consisted of initial denaturation at 98^°^C for 1 min, followed by 30 cycles of denaturation at 98^°^C for 10 s, annealing at 50^°^C for 30 s, and elongation at 72^°^C for 30 s, and extension at 72^°^C for 5 min. The same volume of 1X loading buffer (contained SYB green) was mixed with the PCR products, and electrophoresis was performed on 2% agarose gel for detection. PCR products were mixed in equidensity ratios followed by purification with the Qiagen Gel Extraction Kit (Qiagen, Germany).

Sequencing libraries were generated using TruSeq DNA PCR-Free Sample Preparation Kit (Illumina, United States) following the manufacturer’s recommendations, and index codes were added. The library quality was assessed on the Qubit 2.0 Fluorometer (Thermo Scientific) and Agilent Bioanalyzer 2100 system (Agilent Technologies, United States). Finally, the library was sequenced on an Illumina NovaSeq platform, and 250 bp paired-end reads were generated.

### The processing of 16S rDNA sequencing data

The 16S rDNA sequences were processed using QIIME v.1.9.1 (Caporaso et al., 2010b), USEARCH v.10.0 (Edgar, 2010), and in-house scripts. Paired-end Illumina reads were checked by FastQC v.0.11.5 (de Sena Brandine and Smith, 2019) and processed in the following steps by USEARCH: joining of paired-end reads and relabeling of sequencing names (-fastq_mergepairs), removal of barcodes and primers (-fastx_truncate), filtering of low-quality reads (-fastq_filter), and finding non-redundancy reads (-fastx_uniques). In operational taxonomic units (OTUs), sequence variation is lost because sequences are collapsed, usually at a sequence identity of 97% (Belk et al., 2018). In the present study, unique sequences were denoised, a set of amplicon sequence variants (ASVs) was generated, and chimeras were automatically filtered using the unoise3 algorithm. The bacterial taxonomy assignment was performed using the Ribosomal Database Project (RDP) database and classifier (Wang et al., 2007). Sequences filtered out of the 16S rDNA data set included those assigned to chloroplasts and mitochondria. These representative sequences were further aligned using PyNAST (Caporaso et al., 2010a) with the Greengenes core-set alignment template. Data from each sample were homogenized with the standard of the least amount of data in the samples. Subsequent analysis of alpha diversity and beta diversity were all performed based on this output homogenized data.

### Bioinformatics analysis

All statistical analyses were performed in the R environment (v4.1.1).^1^ The indexes (i.e., Shannon, Chao 1, ACE, Richness, and Invsimpson) of alpha (α) diversity were calculated using the R package “vegan”,^2^ while the microbial beta diversity was estimated according to the unweighted UniFrac distance between the samples using the distance() function in the “phyloseq” package (McMurdie and Holmes, 2013). Comparisons of alpha diversity between drowning and postmortem submersion were performed using a two-sided Wilcoxon rank-sum test, and among PMSIs were calculated using the Kruskal-Wallis (K-W) test. The results of beta diversity were visualized using principal coordinates analysis (PCoA) plots. The unweighted Unifrac distance between the drowning and postmortem submersion and among PMSIs (PERMANOVA) was compared by the adonis2() function in the vegan package.

The proportion of taxa derived from water to the total taxa of gut samples was calculated by fast expectation-maximization microbial source tracking (FEAST) (Shenhav et al., 2019). Samples taken from the water were assigned as sources, whereas animal samples were assigned as sinks. The characterization of microorganismal features differentiating specific PMSI was performed using the linear discriminant analysis (LDA) effect size (LEfSe) method,^3^ which was based on an LDA score > 3.0 and *p* < 0.05. Phylogenetic Investigation of Communities by Reconstruction of Unobserved States (PICRUSt) was used to predict function from the 16S sequence data (Langille et al., 2013). The PICRUSt predictions were categorized as levels 1–3 into KEGG pathways. STAMP software^4^ was used to visualize the predicted functions within the KEGG pathways. Additionally, the phenotype of the microbiome including gram-negative vs. gram-positive, biofilm formation, mobile element content, oxidative stress tolerance, pathogenic potential, and oxygen utilizing was predicted using BugBase with the default parameters (Ward et al., 2017; Estaki et al., 2020).

To prove that the microbial community in the enteric contents could be utilized for PMSI estimation, the relative abundances of bacterial taxa at the different levels were regressed against PMSI using default parameters of the R implementation of the RF algorithm (R package “randomForest”; ntree = 1,000; using default mtry of p/3, where p is the number of taxa). Mean absolute error (MAE) was used to measure the viability of the regression model. Moreover, to identify the taxa serving as biomarkers, the built-in rfcv() function from the “randomForest” package was used to explore the relationship between taxa number and error. A nested cross-validation procedure was implemented to select an optimal predictor number. The number corresponding to the minimum error was considered as an optimal biomarker number. In addition, variables’ importance was analyzed using the percentage increase in the mean-squared error (%IncMSE). A larger%IncMSE denotes that a variable is more important (Liaw and Wiener, 2002).

## Results

### Bacterial community structure overview

Intestinal contents were collected from the exploratory and validation experiment at indicated PMI, as listed in Table 1. Fresh water samples were additionally obtained. The bacterial community profile of each sample was generated by 16S rDNA gene amplification targeting the V3–V4 region, followed by Illumina sequencing. A total of 7,004,470 high-quality reads were obtained after quality control and grouped into 3,144 ASVs among the 185 samples.

**FIGURE 1:**
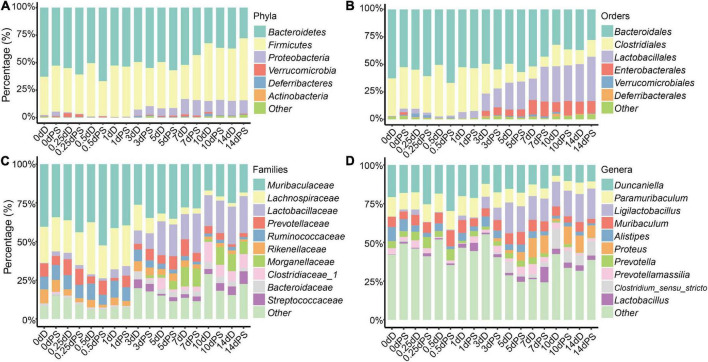
Relative abundance of bacterial taxa at different taxonomic levels. Stacked bar charts of the top 6 bacterial phyla **(A)**, top 6 bacterial orders **(B)**, top 10 bacterial families **(C)**, and top 10 bacterial genera **(D)** with the largest mean relative abundance in the gut. D, drowning group; PS, postmortem submersion group.

Rarefaction curves showed a diminishing rate of new ASV identification with the increase in sequencing depth, implying a good sequence coverage (Supplementary Figure 1). Based on the samples of the exploratory experiment, apparent changes in the intestinal flora were observed in the time points greater than 3 days vs. those within 1 day (Figure 1). At the phylum level, *Bacteroidetes, Firmicutes*, and *Proteobacteria* were the most dominant phyla in both the drowning and postmortem submersion samples (>90%). The abundance of *Proteobacteria* increased in 3 d samples and reached its plateau on 7 d and beyond. *Bacteroidetes* had a higher relative abundance in the early phase than in the late stage of decomposition (Figure 1A). At the order level, the relative abundances of *Lactobacillales* and *Enterobacterales* increased from 3 d and became stable after 7 d, while the relative abundance of *Clostridiales* decreased in 3 d and 7 d samples and then turn overed (Figure 1B). At the family level, *Lactobacillaceae* increased gradually over time and remained high thereafter, whereas abundance of *Lachnospiraceae* and *Ruminococcaceae* declined from 3 d (Figure 1C). At the genus level, the abundance of *Ligilactobacillus* was elevated from 3 d and persisted at a high level. *Proteus* emerged on 3 d and grew until 7 d. The abundance of *Clostridium_sensu_stricto* in the late phase was higher than that in the early stage (Figure 1D). Furthermore, no discernible variations in the intestinal microbiota composition were detected between drowning and postmortem submersion corpses.

**FIGURE 2:**
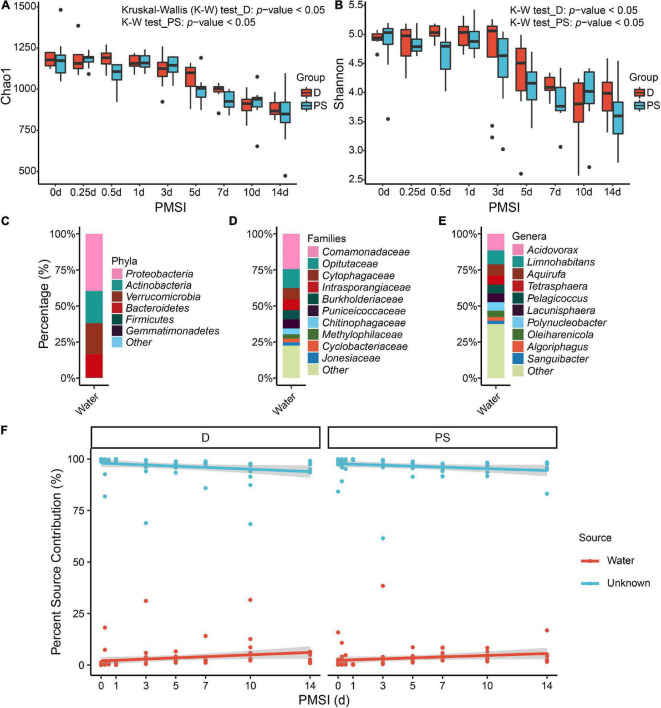
Alpha diversity analysis and microbial source tracking of the intestinal flora. Chao1 **(A)** and Shannon **(B)** are reported, with no difference between the D and PS groups. Stacked bar charts of the top 6 bacterial phyla **(C)**, top 10 bacterial families **(D)**, and top 10 bacterial genera **(E)** with the largest mean relative abundance in water samples. **(F)** Percentage source contribution of water microbial communities to intestinal microbial communities over time determined using FEAST. The percentage source contribution is listed on the y-axis, and PMSI is listed on the x-axis. Displayed is the percentage source contribution of water microbial communities to the intestinal flora for each PMSI, as well as the percentage source contribution of unknown microbial communities. Red indicates the contribution of water, while blue indicates the unknown source contribution for microbiomes. The red and blue lines represent the fitted regression line for contributions of water and unknown source, respectively. The shaded regions indicate 95% credible intervals. D, drowning group; PS, postmortem submersion group.

Alpha diversity was further performed using samples from the exploratory experiment. The Chao 1 and the Shannon indices were decreased over time, while no significant difference was observed between the drowning and postmortem submersion subgroups at the same time points (Figures 2A,B and Table 2). In line with this, similar results were disclosed by ACE, Richness, and Invsimpson indices (Supplementary Figures 2A–C and Supplementary Table 1). Then, bacterial communities of the fresh water were analyzed. Though there were some phyla overlap between water and gut, none of the top 10 families and genera was shared (Figures 2C–E). Considering that the water-derived bacteria might enter the bloodstream and take effect on the composition of the gut flora during drowning, FEAST analysis was conducted to trace the intestinal microbiota origin in both the drowning and postmortem submersion subgroups. Unexpectedly, there was no contribution of water-derived bacteria to the gut microbiome in the drowning subgroup at postmortem 0 h (Figure 2F). The intestinal microbial community components exhibited a PMSI-dependent pattern both in the drowning and postmortem submersion subgroups, but with less impact from the cause of death (Figure 2F). Thus, data from the two subgroups were subsequently assessed to trace the common bacterial community succession.

**TABLE 2:** Comparisons of alpha diversity indexes (Chao1 and Shannon) between drowning and postmortem submersion by Wilcoxon rank-sum test.

PMSI	*p*_ Chao1	*p*.adjust_ Chao1*	*p*_ Shannon	*p*.adjust_ Shannon*
0 d	0.721	0.898	0.328	0.422
0.25 d	0.798	0.898	0.721	0.721
0.5 d	0.010	0.094	0.007	0.063
1 d	0.959	0.959	0.442	0.497
3 d	0.442	0.898	0.105	0.422
5 d	0.234	0.702	0.328	0.422
7 d	0.038	0.171	0.161	0.422
10 d	0.645	0.898	0.279	0.422
14 d	0.574	0.898	0.234	0.422

*P-values were adjusted using Benjamini-Hochberg (BH) correction and the adjusted p-value cut-off was 0.05.

### Temporal succession patterns of the intestinal microbial communities

**FIGURE 3:**
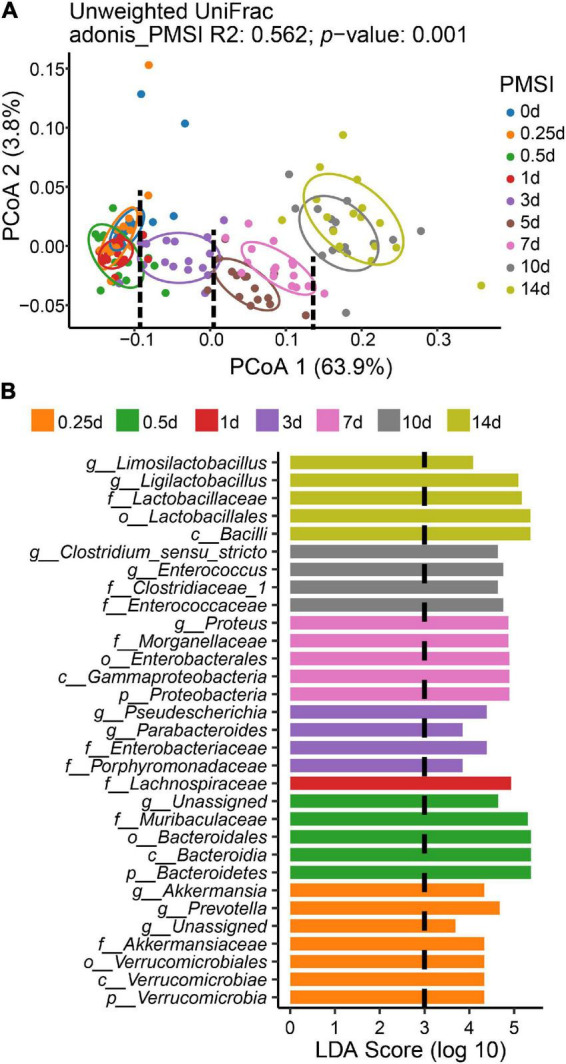
Beta diversity analysis and LEfSe comparison of the intestinal flora. **(A)** Ordination plot for the first two PCoA axes based on Unweighted Unifrac distance. Beta diversity differed significantly among PMSIs (PERMANOVA, *R*^2^ = 0.562, *p* = 0.001). The circle showed a 68% confidence interval. **(B)** Comparison of microbial variations among PMSIs using LEfSe analysis.

To measure the dissimilarity in postmortem microbial communities among different PMSIs, unweighted UniFrac-based PCoA was applied and exhibited in a two-dimensional space (Figure 3A). The result demonstrated that samples clustered based on PMSI (PERMANOVA, *R*^2^ = 0.562, *p* = 0.001). The first axis (PCoA1) separated the communities mainly by PMSI and explained 63.9% of the variance, and the second axis (PCoA2) explained 3.8% of the variance. Samples were separated and broadly classified into four categories: within 1, 3, 5–7, and 10–14 days. LEfSe analysis revealed certain bacteria indicative of PMSI (Figure 3B and Supplementary Figure 3). At the genus level, high abundance of *Pseudescherichia* and *Parabacteroides* were relevant to 3 d. The greatest increase in *Proteus* pointed to 7 d. 10 d was characterized by a higher abundance of *Enterocoecus* and *Clostridium_sensu_stricto*. The genus *Ligilactobacillus* and *Limosilactobacillus* were noteworthy at 14 d.

**FIGURE 4:**
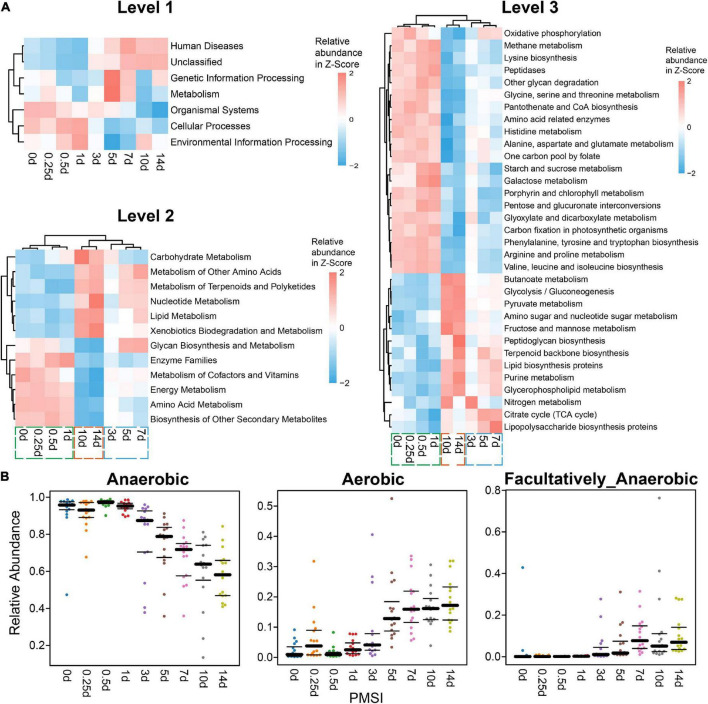
Functional prediction analysis and phenotypic prediction based on bacterial sequences. **(A)** The heatmaps of the normalized relative abundance of the imputed functional profiles using PICRUSt grouped into levels 1–3 functional categories. At level 2, only significantly changed functions (*p.*adjust < 0.05) were shown. At level 3, only significantly changed functions (*p.*adjust < 0.05) with the relative abundance higher than 1% were shown. **(B)** Discrepancy in the gut microbial community phenotypes associated with aerobic, anaerobic, and facultatively anaerobic bacteria among PMSIs during decomposition.

Additionally, functions and phenotypes of intestinal microbial community were further assessed via PICRUSt and BugBase analysis, respectively. Based on KEGG orthology, PCA demonstrated that samples clustered in two main categories (within 3 days and beyond 3 days) (Supplementary Figure 4). Functions of gut microbiota were stable during the initial corruption stage, which was changed significantly in the mid-late decomposition stages (Supplementary Figure 4). At KEGG level 1, metabolism accounted for > 48% of all predicted function (Supplementary Table 2), among which the top 3 pathways were carbohydrate, amino acid, and energy metabolism at level 2 (Supplementary Table 3). Most genes belonged to purine metabolism, peptidases, amino sugar and nucleotide sugar metabolism, and amino acid-related enzymes at level 3 (Supplementary Table 4). As shown in the heatmaps (Figure 4A), various pathways exhibited temporal changes with PMSI. Based on the distributions of significantly different functional pathways (differential pathways related to microbial metabolism were selected at level 2 and 3) among PMSIs, the gut flora during 14 days of decomposition could be clustered into three periods (within 1, 3–7, and 10–14 days). Additional BugBase analysis revealed that phenotypes of the intestinal microbial community also exhibited a temporal variation. Gut flora was mainly composed of anaerobic bacteria at the initial stage of decomposition, and then gradually transferred to aerobic and facultative anaerobic bacteria after 7 days (Figure 4B).

### Postmortem submersion interval estimation based on intestinal microbiota

**FIGURE 5:**
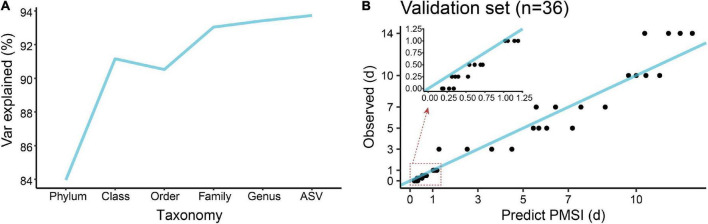
Regression model establishment for PMSI estimation based on the random forest algorithm. **(A)** Comparison of model fit at different taxonomic levels. **(B)** Predicted vs. actual PMSI of the validation samples obtained by the regression model established on all genera. The blue line represents the y = x diagonal line. The dots represent the samples from the validation experiment (*n* = 4 per PMSI). The inserted graph represents the results of samples obtained within 1 day.

In terms of community composition, diversity, functions, and phenotypes, there was a clear change in succession of the gut bacterial communities. To verify whether the succession could be used to estimate PMSI in the aquatic environment, we established regression models based on intestinal microbial community. RF, a robust and superior algorithm in accuracy in benchmarking tests on microbiome data sets (Knights et al., 2011), were used in the present study. The relative abundance of gut bacteria at the phylum, class, order, family, genus, and ASV levels from the exploratory experiment were regressed against PMSI. The model based on the microbiota information at the ASV level showed the best-fitting result (93.73% Var explained, 93.73% of the gut microbiota variance related to PMSI was explained) among all taxonomic levels (Figure 5A). Similar performance was observed at the genus level (93.42% variation explained). The regression model based on various genera (hereafter called the full model) obtained a satisfactory performance for the data from exploratory experiment [MAE (± SE) = 0.763 (± 0.073) d]. Then, the full model was further verified in samples from the independent validation experiment, which presented an excellent predictive ability [MAE (± SE) = 0.750 (± 0.142) d, Figure 5B and Table 3)]. Furthermore, we considered the prediction results of samples that obtained within 1 day postmortem. The MAEs (± SE) of the exploratory experiment and validation experiment were 0.302 (± 0.064) d and 0.162 (± 0.023) d, respectively. Performances of the model were similar for exploratory and validation data, indicating that the model were not over-fit. These results suggested that gut microbiota could be used for estimating PMSI in submerged corpses.

**TABLE 3:** Prediction results of validation samples derived from the full and simplified regression models.

Sample	Group	Observed*	Predict_ full*	Error_ full*	Predict_ simplified*	Error_ simplified*
V1	D	0	0.185	0.185	0.163	0.163
V2	D	0	0.339	0.339	0.467	0.467
V3	D	0.25	0.398	0.148	0.221	–0.029
V4	D	0.25	0.315	0.065	0.417	0.167
V5	D	0.5	0.616	0.116	0.531	0.031
V6	D	0.5	0.545	0.045	0.700	0.200
V7	D	1	1.143	0.143	0.919	–0.081
V8	D	1	1.055	0.055	0.751	–0.249
V9	D	3	2.526	–0.474	2.615	–0.385
V10	D	3	1.279	–1.721	1.340	–1.660
V11	D	5	5.452	0.452	5.632	0.632
V12	D	5	6.042	1.042	6.660	1.660
V13	D	7	5.576	–1.424	5.547	–1.453
V14	D	7	6.433	–0.567	6.275	–0.725
V15	D	10	9.660	–0.340	9.917	–0.083
V16	D	10	10.002	0.002	9.956	–0.044
V17	D	14	10.376	–3.624	9.705	–4.295
V18	D	14	11.947	–2.053	12.448	–1.552
V19	PS	0	0.202	0.202	0.488	0.488
V20	PS	0	0.277	0.277	0.300	0.300
V21	PS	0.25	0.358	0.108	0.372	0.122
V22	PS	0.25	0.527	0.277	0.904	0.654
V23	PS	0.5	0.722	0.222	0.559	0.059
V24	PS	0.5	0.694	0.194	0.790	0.290
V25	PS	1	1.191	0.191	1.066	0.066
V26	PS	1	1.021	0.021	0.815	–0.185
V27	PS	3	3.611	0.611	3.379	0.379
V28	PS	3	4.502	1.502	4.333	1.333
V29	PS	5	7.181	2.181	7.747	2.747
V30	PS	5	5.693	0.693	5.643	0.643
V31	PS	7	7.555	0.555	7.530	0.530
V32	PS	7	8.630	1.630	9.270	2.270
V33	PS	10	10.431	0.431	9.958	–0.042
V34	PS	10	11.037	1.037	10.913	0.913
V35	PS	14	11.428	–2.572	11.032	–2.968
V36	PS	14	12.481	–1.519	12.414	–1.586

*Predict_full and Error_full: Prediction results of validation samples derived from the regression model based on all genera. Predict_simplified and Error_simplified: Prediction results of validation samples derived from the regression model established by 15 identified genera. PMSI was measured in units of days.

**FIGURE 6:**
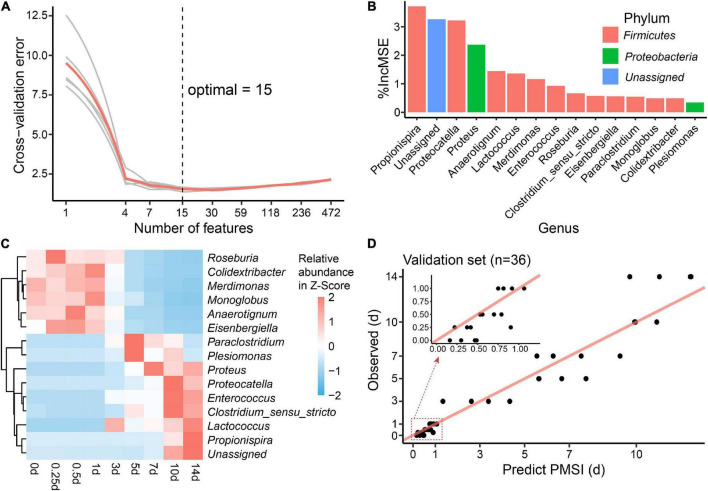
Biomarker identification and simplified model establishment for PMSI estimation. **(A)** Ten-fold cross-validation result of the full regression model. **(B)** The top 15 biomarker bacterial genera were identified by the random forest algorithm. Biomarker taxa were ranked in decreasing order of importance (i.e.,%IncMSE). **(C)** Heatmap showing the relative abundances of the top 15 PMSI-predictive biomarker bacterial genera. **(D)** Predicted vs. actual PMSI of the validation samples obtained by the simplified regression model established on the identified genera. The red line represents the y = x diagonal line. The dots represent the samples from the validation experiment (*n* = 4 per PMSI). The inserted graph represents the results of samples obtained within 1 day.

Additionally, cross-validation showed that the predicted error decreased sharply with the increase in genus number until it exceeded 15 (Figure 6A), which implied that a better simplified model would be reestablished based on refined genera. First, 15 bacterial taxa at the genus level were selected according to their time-discriminatory importance (Figures 6B,C). Of the 15 candidate genera, 12 were from *Firmicutes* phylum, 2 belonged to *Proteobacteria*, and 1 was unassigned. Some biomarker taxa, such as *Roseburia, Merdimonas*, and *Monoglobus*, showed high relative abundance in the corresponding initial decomposition stage and decreased over the PMSI. Others including *Proteus, Enterococcus*, and *Lactococcus* started to accumulate at 3–5 days postmortem and remained at high levels in the late decomposition stage (Figure 6C). Then, based on the selected genera, an optimized RF regression model (hereafter called the simplified model) was reconstructed for PMSI estimation, which presented a MAE (± SE) of 0.701 (± 0.071) d for the exploratory experiment data and 0.818 (± 0.165) d for the validation experimental data (Figure 6D and Table 3). When it came to samples decomposed within 1 day, the MAEs (± SE) were 0.294 (± 0.063) d and 0.222 (± 0.045) d, respectively. These results further illustrated that the time-dependent change of selected genera was a potential tool for PMSI estimation, which merits further study.

## Discussion

Microbiome tools have recently been proposed as a potential approach for PMI or PMSI estimation in forensic investigations (Oliveira and Amorim, 2018). However, there remains a paucity of knowledge concerning intestinal postmortem microbial community succession in corpses recovered from water. In this study, exploratory analysis of gut bacteria was performed using the 16S rDNA sequencing approach in mice decomposing in natural aqueous settings. Temporal succession of gut flora was disclosed during the 14 days degradation period, which was relatively independent of the cause of death. Furthermore, based on the relative abundance of the microbiota, a regression model was established and exhibited satisfactory performance for PMSI estimation. Our study presented the potential use of gut flora succession for forensic investigation on corpses recovered from water.

Microbial community stability has been documented up to 2 days after death in terrestrial research (Pechal et al., 2018). We observed that the intestinal microbes of drowning or postmortem submersion corpses were stable within 1 day, which were almost completely represented by normal bacterial flora of the intestinal tract. Unlike the obvious differences in microbial communities of internal organs/tissues (i.e., lung, liver, blood) between drowning and postmortem submersion corpses (Wang et al., 2020), the differences in gut flora were not observed during the 14 days decaying period, demonstrating the higher stability of the gut microbiome under different causes of death. Although microorganisms could enter the digestive tract with water during drowning, their proportion in the whole gut flora was minimal, which would be explained by the fact that water-derived bacterial species could not colonize and reproduce rapidly due to the distinct environmental differences in the gut. With postmortem degradation, the rapid shifting of the gut environmental conditions (e.g., pH) can significantly influence the destination of the microbiome. A previous study reported that increased competition in the microbial communities occurs postmortem, especially in the later phase of decomposition (Pechal et al., 2018). It might cause the richness and diversity of the microbial community to decrease with the decomposition process, similar to porcine remains in aquatic systems (Kaszubinski et al., 2020) and mouse corpses in terrestrial systems (Liu et al., 2021). In our present study, the shift in intestinal microbial communities was more pronounced in 3–7 days than in 10–14 days, suggesting that the microbial succession slowed down in the later stages of decomposition as previously reported (Belk et al., 2018).

The variations in microbial communities are accompanied by changes in function and phenotype. In this study, microbial gene functions related to metabolic pathways such as energy metabolism, amino acid metabolism, and enzyme families were significantly higher within 1 day, which represented the normal function of the gut. However, at the late phase of decomposition, the microbial gene functions showed the increased ability on lipid metabolism and nucleotide metabolism. Studies have related the increased adipocere formation to the presence of *Clostridium* (e.g., *Firmicutes, Clostridia*, and *Clostridiaceae*) in warm anaerobic environments, due to the production of lecithinase (Ralebitso-Senior, 2018). In the future, combining various types of microbiome data, such as DNA sequence data and metabolomics, with the cadaveric phenomena might enhance our understanding of the degradation process. According to the analysis of bacterial phenotypes, > 90% of the intestinal flora was made up of anaerobes due to the physical condition within 1 day postmortem. After 3 days, facultative anaerobes and aerobic bacteria significantly increased instead of anaerobic bacteria. This might have resulted from a phenomenon that the corpses floated to the surface and were exposed to air at 1–2 days after death. Additionally, air might enter the gastrointestinal tract accompanied by some water during decomposition.

Microbiome community succession is attributed to the relative abundance and species in the microbiota. In the aquatic system, several predominant families in healthy gut, such as *Muribaculaceae, Ruminococcaceae*, and *Lachnospiraceae*, declined after 3 days postmortem, which is consistent with the results from both human and mouse remains in the terrestrial environment (DeBruyn and Hauther, 2017; Liu et al., 2021). *Clostridiaceae* and *Clostridium* have been reported to be a key contributor to decomposition in both terrestrial (Metcalf et al., 2013; Pechal et al., 2014; Hyde et al., 2015) and aquatic (Cartozzo et al., 2021b) settings, which is in line with our present study. At the genus level, *Proteus* and *Enterococcus* were the most predominant bacteria of remains decaying in the terrestrial environment after 3 days postmortem (Janaway et al., 2009; Li et al., 2021). *Proteus* might be outcompeting other bacteria at later PMSIs due to the swarming behavior (Kearns, 2010). In the present study, the results supported the specific bacteria changes in the aquatic environment and their applicability as indicators for PMSI estimation. The similar change patterns of the above-mentioned bacteria could be observed in terrestrial and aquatic putrefaction processes, indicating that they were less affected by the environment and warranted further investigation. Meanwhile, the slight increase in *Lactobacillus* found in our study was contrary to the carcasses degraded in land (DeBruyn and Hauther, 2017; Li et al., 2021). Though the colonized intestinal microbiome is stable, the water-derived bacteria taxa were elevated slightly in the whole intestinal microbiome over the PMSI, indicating the possible impact of water on longer-term decomposition. Metcalf et al. characterize the microbial communities on the skin, abdominal cavity, and gravesoil associated with mice corpse during decaying (Metcalf et al., 2016). They reveal the decomposer community is derived primarily from bulk soil. In conjunction with our findings, these results suggest that microorganisms in the surroundings have an important role in decomposition progress. More specific studies are worth carrying out on corpses recovered from water.

Low-cost, high-throughput technologies allow us to accumulate molecular data quickly and to apply sophisticated machine-learning algorithms, building generalizable predictive models that will be useful in the criminal justice system (Metcalf et al., 2017). The capacity of machine-learning algorithms [such as RF, support vector machine (SVM), and artificial neural network (ANN)] to generalize and analyze complex non-linear relationships make them suitable to model the relationships between microbial community succession and PMI or PMSI. For instance, RF has been used to establish models based on gut microbiota to estimate PMI in mice carcasses in terrestrial habitats (Liu et al., 2020, 2021). It is known that the performance of SVM and ANN is sensitive to the adjustment of parameters (such as the penalty parameter C and the kernel parameter γ for SVM) (Ma et al., 2014). Additionally, it is easy to make the prediction accuracy fall into a local optimum for ANN (Huang and Liu, 2019). RF is an ensemble algorithm that builds randomized decision trees and incorporates a variety of features into its classification or regression process (Breiman, 2001). It has crucial advantages relative to other algorithms, including rapid training speed, tuning simplicity, and robustness to noise. Importantly, it can deter overfitting by a “majority voting” approach (Lebedev et al., 2014). Thus, based on the succession of gut flora, we built regression models of PMSI estimation using RF for freshwater submersed corpses in the present study. The predicted performance was satisfactory for PMSI estimation during the 14 days decomposition both in the original model [MAE (± SE) = 0.750 (± 0.142) d, approximately 18 h] and simplified model [MAE (± SE) = 0.818 (± 0.165) d, approximately 19.632 h] obtained. Wallace et al. (2021) examined the succession of bacteria colonized on the external surface of porcine carcasses submerged in a tidal-influenced river over 19 days of decomposition and obtained an RF model (77.2% variation explained) using the various genera. Meanwhile, our model based on the intestinal microbiome explained 93.42% of the variation, which demonstrates that the succession of the gut flora might be better than the external surface microbiome for PMSI estimation. In addition, it is noteworthy that most of the selected genus for PMSI estimation were not the dominant taxa in the gut microbiota postmortem, which showed a certain succession pattern and belonged to the phyla *Firmicutes* and *Proteobacteria*. Furthermore, the errors in PMSI estimation increased significantly for the 14 days samples, which might be attributed to the fact that decomposition is not entirely deterministic and is subject to stochasticity that would result in slightly different trajectories of the bacterial communities between individuals (DeBruyn and Hauther, 2017). However, there are still existing gaps between the current research and its practical application. First, different microbial communities may occur in different aquatic ecosystems (e.g., flowing or non-flowing habitats, seasons) (Lang et al., 2016; Metcalf et al., 2016) and contribute to the succession of the submersed intestinal microbiome. Second, the PMSI model based on animal experiments is hard to validate on human remains, because the gut microflora of humans is likely to be affected by the complex internal or external environments, such as variations in the diet and medication history. The impact of different conditions on PMSI estimation and its application in forensic practice is still worth exploring. Finally, model performance would be influenced by experimental design, the characteristics of the dataset (such as the number of features, and sample size), and the properties of different machine learning algorithms (e.g., strategy for statistical analysis) (Liebal et al., 2020). Comparison of the applicability among different algorithms for microbiome data should also be included in future studies.

Postmortem succession of gut microbiome has been shown to be a stable and reliable biomarker for PMI in the terrestrial environment both in animal models (Liu et al., 2020, 2021) and human remains (Hauther et al., 2015; DeBruyn and Hauther, 2017). Considering the fluctuations in surrounding biological and environmental conditions (e.g., weather, insect accessibility and cadaver position), the succession of the microbial community associated with submerged bones or colonized on the external surfaces of corpses might be largely affected in relation to PMSI estimation (Cartozzo et al., 2021a,b; Wallace et al., 2021). In this study, we provided evidence that gut microbial succession is stable and has a valuable potential for PMSI estimation, providing a new insight into our overall understanding of the decomposition processes of submersed corpses.

## Data availability statement

The data presented in this study are deposited in the NCBI SRA BioProject repository, accession number: PRJNA856392.

## Ethics statement

The animal study was reviewed and approved by the Animal Experiment Committee of China Medical University.

## Author contributions

RZ, LW, and DG conceived and designed the research. FZ and PW performed the lab experiments and wrote the main manuscript text. ZW, XL, HY, and SD performed the animal experiments. FZ, KZ, and HY performed the bioinformatic analysis. All authors have read and commented on the manuscript.

## Footnotes

1
http://www.r-project.org/

2
https://cran.r-project.org/package=vegan

3
http://huttenhower.sph.harvard.edu/lefse/

4
http://kiwi.cs.dal.ca/Software/STAMP
