# Supplementary material for: Postmortem submersion interval estimation of cadavers recovered from freshwater based on gut microbial community succession
Source: Front Microbiol. 2022 Dec 2;13:988297. doi: 10.3389/fmicb.2022.988297 (PMC9756852; doi:10.3389/fmicb.2022.988297)
Supplement: Supplementary file 1 [file Data_Sheet_1.docx]

Supplementary Material

# Supplementary Figures and Tables

## Supplementary Figures


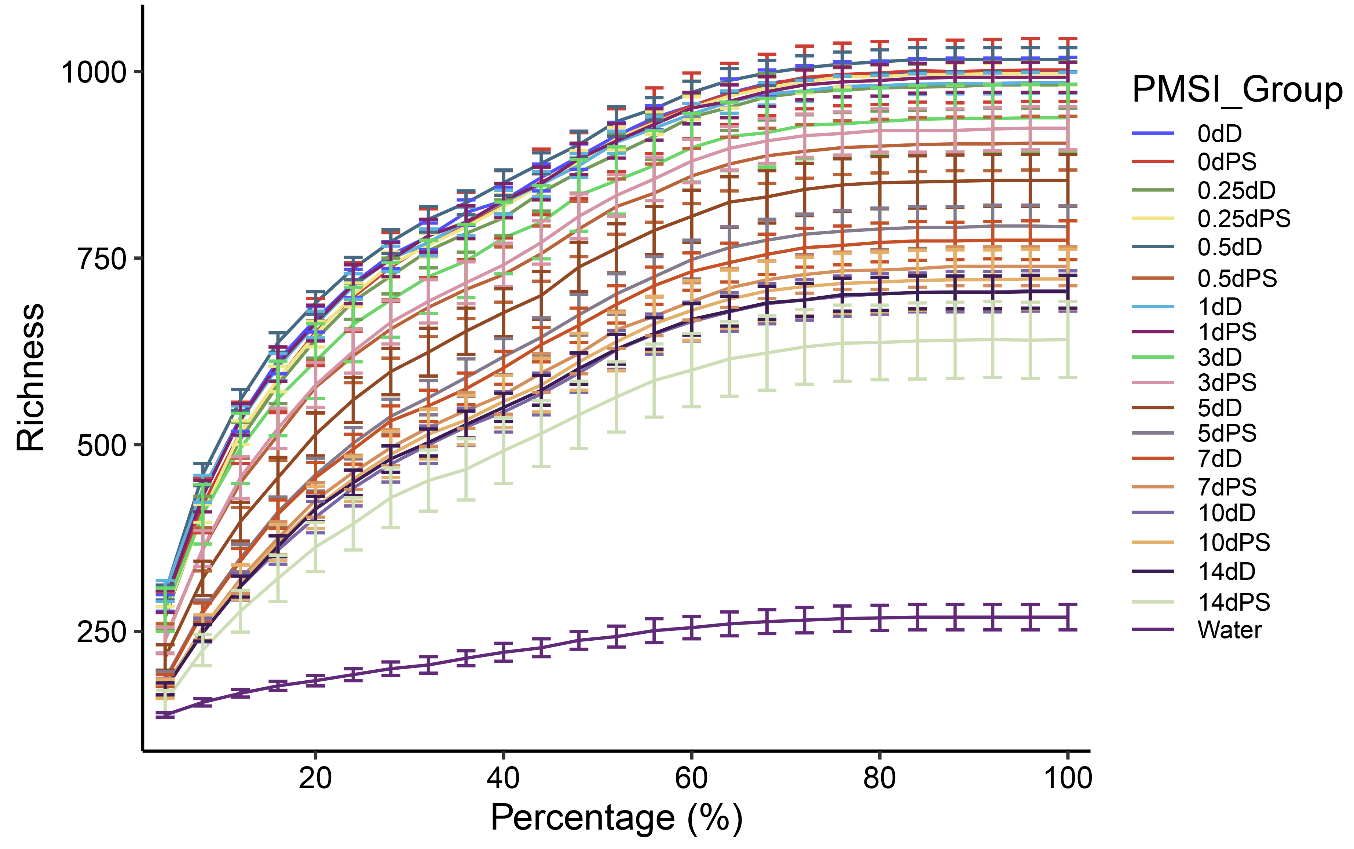


**Supplementary Figure 1.** Rarefaction curves of detected bacterial ASVs of the gut microbiota with increasing sequencing depth. Vertical bar represents the standard error.


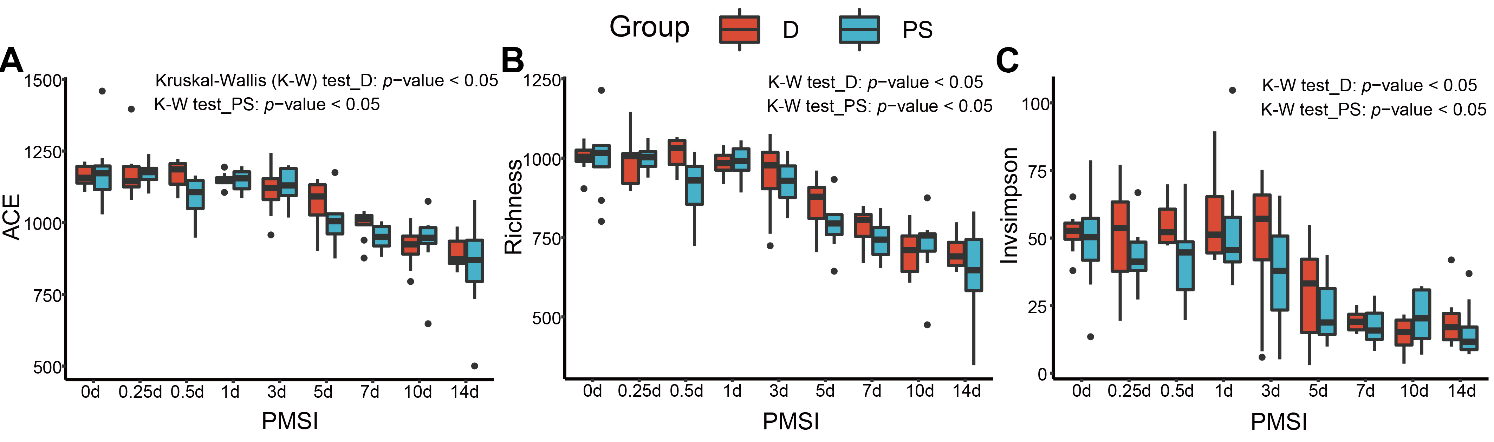


**Supplementary Figure 2.** Comparisons of the ACE (**A**), Richness (**B**), and Invsimpson (**C**) indices among different PMSIs and between drowning and postmortem submersion groups. The indices were significantly reduced over time during decomposition, with no difference between the drowning and postmortem submersion groups. D, drowning group; PS, postmortem submersion group.


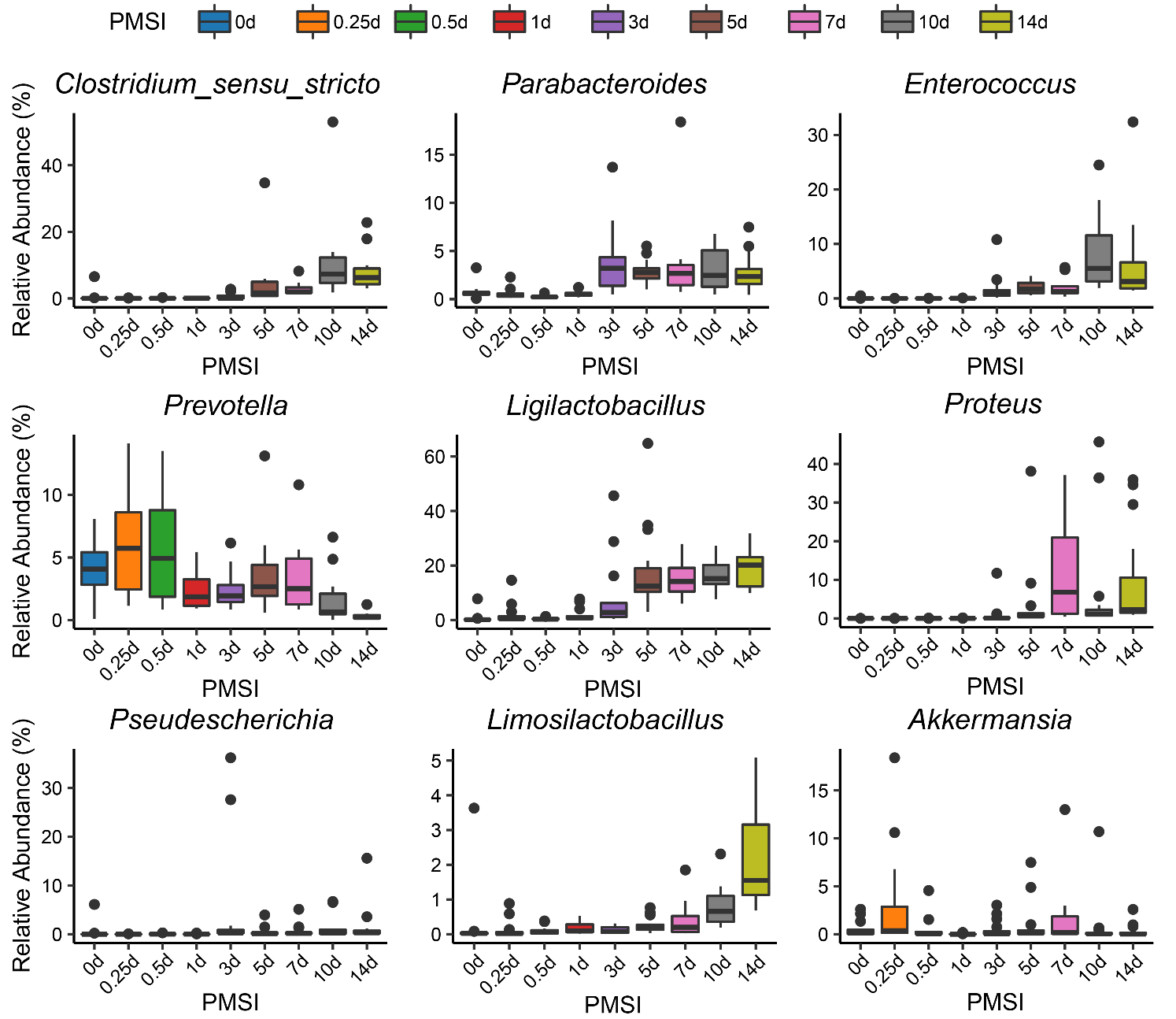


**Supplementary Figure 3.** Changes in relative abundance during decomposition for genera selected by LEfSe.


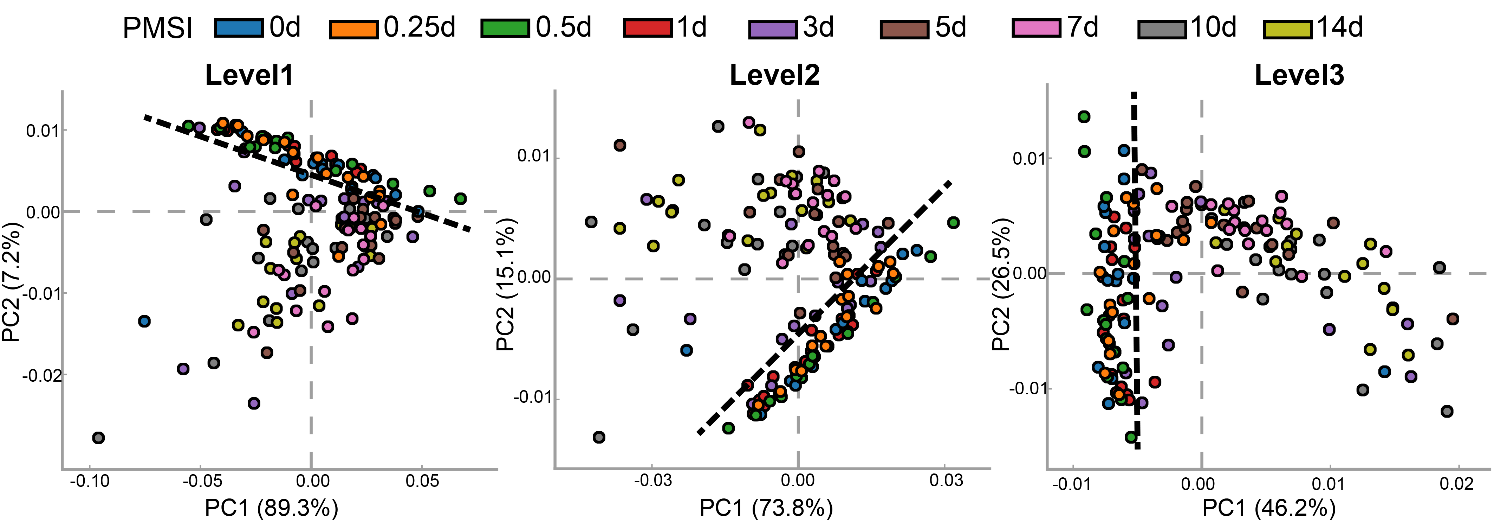


**Supplementary Figure 4.** PCA plots produced by STAMP analysis of PICRUSt-projected functional profiles (level 1–3, KEGG orthology).

## Supplementary Tables

**Supplementary Table 1** Comparisons of alpha diversity indexes (ACE, Richness, and Invsimpson) between drowning and postmortem submersion by Wilcoxon rank-sum test

| PMSI | *p*_ACE | *p*.adjust_ACE | *p*_Richness | *p*.adjust_Richness | *p*_Invsimpson | *p*.adjust_Invsimpson |
| --- | --- | --- | --- | --- | --- | --- |
| 0d | 1.000 | 1.000 | 0.645 | 0.829 | 0.645 | 0.645 |
| 0.25d | 0.721 | 1.000 | 0.798 | 0.834 | 0.442 | 0.645 |
| 0.5d | 0.021 | 0.171 | 0.016 | 0.140 | 0.050 | 0.449 |
| 1d | 0.959 | 1.000 | 0.834 | 0.834 | 0.574 | 0.645 |
| 3d | 0.798 | 1.000 | 0.574 | 0.829 | 0.161 | 0.527 |
| 5d | 0.234 | 0.702 | 0.161 | 0.516 | 0.505 | 0.645 |
| 7d | 0.038 | 0.171 | 0.172 | 0.516 | 0.442 | 0.645 |
| 10d | 0.442 | 0.995 | 0.442 | 0.796 | 0.234 | 0.527 |
| 14d | 0.645 | 1.000 | 0.328 | 0.738 | 0.234 | 0.527 |

* P-values were adjusted using Benjamini-Hochberg (BH) correction and the adjusted *p* value cut-off was 0.05

**Supplementary Table 2** Predicted functions at level 1 of the gut bacterial microbiota during decomposition

| Functions (%) | 0d | 0.25d | 0.5d | 1d | 3d | 5d | 7d | 10d | 14d | *p*.adjust |
| --- | --- | --- | --- | --- | --- | --- | --- | --- | --- | --- |
| Cellular Processes | 3.274 | 3.149 | 3.369 | 3.410 | 2.858 | 2.498 | 2.582 | 2.775 | 2.572 | 0.000 |
| Environmental Information Processing | 12.804 | 12.591 | 13.223 | 13.677 | 12.657 | 11.315 | 11.770 | 13.236 | 12.656 | 0.033 |
| Genetic Information Processing | 19.924 | 20.002 | 19.675 | 19.724 | 19.796 | 20.456 | 20.173 | 19.623 | 20.084 | 0.204 |
| Human Diseases | 0.789 | 0.773 | 0.752 | 0.743 | 0.828 | 0.873 | 0.919 | 0.892 | 0.894 | 0.000 |
| Metabolism | 48.687 | 49.025 | 48.737 | 48.227 | 48.848 | 49.815 | 49.213 | 48.144 | 48.494 | 0.169 |
| Organismal Systems | 0.726 | 0.725 | 0.705 | 0.693 | 0.704 | 0.696 | 0.674 | 0.633 | 0.616 | 0.000 |
| Unclassified | 13.795 | 13.734 | 13.538 | 13.525 | 14.309 | 14.346 | 14.670 | 14.697 | 14.683 | 0.000 |

**Supplementary Table 3** Part of the predicted functions at level 2 of the gut bacterial microbiota during decomposition

| Functions (%) | 0d | 0.25d | 0.5d | 1d | 3d | 5d | 7d | 10d | 14d | *p*.adjust |
| --- | --- | --- | --- | --- | --- | --- | --- | --- | --- | --- |
| Amino Acid Metabolism | 20.264 | 20.251 | 20.235 | 20.120 | 19.730 | 19.470 | 19.356 | 18.968 | 19.026 | 0.000 |
| Biosynthesis of Other Secondary Metabolites | 2.172 | 2.172 | 2.209 | 2.166 | 2.066 | 2.049 | 1.969 | 1.864 | 1.849 | 0.000 |
| Carbohydrate Metabolism | 21.337 | 21.299 | 21.626 | 21.866 | 21.891 | 21.435 | 21.355 | 22.528 | 22.178 | 0.002 |
| Energy Metabolism | 12.254 | 12.278 | 12.239 | 12.193 | 12.037 | 12.095 | 12.020 | 11.733 | 11.682 | 0.000 |
| Enzyme Families | 4.682 | 4.645 | 4.723 | 4.759 | 4.547 | 4.492 | 4.517 | 4.471 | 4.447 | 0.000 |
| Glycan Biosynthesis and Metabolism | 6.327 | 6.405 | 6.374 | 6.169 | 6.279 | 6.569 | 6.585 | 5.929 | 5.900 | 0.001 |
| Lipid Metabolism | 5.721 | 5.757 | 5.633 | 5.687 | 5.826 | 5.890 | 6.039 | 6.166 | 6.186 | 0.000 |
| Metabolism of Cofactors and Vitamins | 8.873 | 8.830 | 8.796 | 8.701 | 8.735 | 8.708 | 8.737 | 8.551 | 8.504 | 0.003 |
| Metabolism of Other Amino Acids | 3.233 | 3.200 | 3.207 | 3.205 | 3.264 | 3.334 | 3.384 | 3.348 | 3.371 | 0.000 |
| Metabolism of Terpenoids and Polyketides | 3.342 | 3.357 | 3.240 | 3.243 | 3.452 | 3.543 | 3.554 | 3.595 | 3.624 | 0.000 |
| Nucleotide Metabolism | 8.596 | 8.604 | 8.597 | 8.683 | 8.688 | 8.920 | 8.925 | 8.913 | 9.166 | 0.000 |
| Xenobiotics Biodegradation and Metabolism | 3.200 | 3.202 | 3.120 | 3.208 | 3.484 | 3.495 | 3.559 | 3.934 | 4.068 | 0.000 |

* Only significantly changed functions (*p*.adjust < 0.05) were shown.

**Supplementary Table 4** Part of the predicted functions at level 3 of the gut bacterial microbiota during decomposition

| Functions (%) | 0d | 0.25d | 0.5d | 1d | 3d | 5d | 7d | 10d | 14d | *p*.adjust |
| --- | --- | --- | --- | --- | --- | --- | --- | --- | --- | --- |
| Purine metabolism | 4.560 | 4.584 | 4.541 | 4.588 | 4.689 | 4.853 | 4.902 | 4.949 | 5.081 | 0.000 |
| Peptidases | 4.053 | 4.042 | 4.085 | 4.107 | 3.936 | 3.965 | 3.960 | 3.869 | 3.886 | 0.000 |
| Amino sugar and nucleotide sugar metabolism | 2.999 | 3.032 | 3.095 | 3.131 | 3.104 | 3.151 | 3.103 | 3.282 | 3.276 | 0.000 |
| Amino acid related enzymes | 3.098 | 3.103 | 3.085 | 3.102 | 3.035 | 3.040 | 3.024 | 2.947 | 3.003 | 0.000 |
| Methane metabolism | 2.777 | 2.705 | 2.809 | 2.849 | 2.614 | 2.546 | 2.483 | 2.411 | 2.447 | 0.000 |
| Oxidative phosphorylation | 2.652 | 2.694 | 2.628 | 2.580 | 2.546 | 2.646 | 2.640 | 2.477 | 2.450 | 0.016 |
| Arginine and proline metabolism | 2.648 | 2.646 | 2.675 | 2.651 | 2.522 | 2.384 | 2.358 | 2.323 | 2.296 | 0.000 |
| Glycolysis / Gluconeogenesis | 2.144 | 2.135 | 2.126 | 2.182 | 2.263 | 2.287 | 2.293 | 2.455 | 2.481 | 0.000 |
| Alanine, aspartate and glutamate metabolism | 2.269 | 2.280 | 2.292 | 2.272 | 2.247 | 2.260 | 2.224 | 2.144 | 2.180 | 0.005 |
| Pyruvate metabolism | 2.044 | 2.040 | 2.020 | 2.051 | 2.136 | 2.123 | 2.136 | 2.259 | 2.268 | 0.000 |
| Starch and sucrose metabolism | 2.046 | 2.033 | 2.109 | 2.124 | 2.031 | 1.957 | 1.892 | 1.950 | 1.912 | 0.000 |
| Fructose and mannose metabolism | 1.719 | 1.751 | 1.774 | 1.823 | 1.929 | 1.922 | 1.863 | 2.173 | 2.105 | 0.000 |
| Glycine, serine and threonine metabolism | 1.859 | 1.867 | 1.879 | 1.854 | 1.803 | 1.824 | 1.826 | 1.731 | 1.735 | 0.000 |
| Galactose metabolism | 1.725 | 1.728 | 1.832 | 1.852 | 1.692 | 1.660 | 1.618 | 1.607 | 1.600 | 0.000 |
| Lysine biosynthesis | 1.705 | 1.716 | 1.730 | 1.736 | 1.661 | 1.638 | 1.621 | 1.578 | 1.628 | 0.000 |
| Peptidoglycan biosynthesis | 1.646 | 1.637 | 1.616 | 1.640 | 1.643 | 1.659 | 1.664 | 1.663 | 1.714 | 0.005 |
| Phenylalanine, tyrosine and tryptophan biosynthesis | 1.773 | 1.788 | 1.804 | 1.761 | 1.665 | 1.575 | 1.568 | 1.466 | 1.456 | 0.000 |
| Valine, leucine and isoleucine biosynthesis | 1.560 | 1.580 | 1.595 | 1.582 | 1.486 | 1.402 | 1.398 | 1.354 | 1.361 | 0.000 |
| Citrate cycle (TCA cycle) | 1.437 | 1.431 | 1.377 | 1.340 | 1.476 | 1.530 | 1.525 | 1.472 | 1.432 | 0.005 |
| One carbon pool by folate | 1.458 | 1.464 | 1.458 | 1.443 | 1.396 | 1.432 | 1.396 | 1.294 | 1.327 | 0.000 |
| Nitrogen metabolism | 1.395 | 1.362 | 1.376 | 1.369 | 1.446 | 1.388 | 1.377 | 1.442 | 1.392 | 0.003 |
| Butanoate metabolism | 1.304 | 1.273 | 1.266 | 1.301 | 1.356 | 1.343 | 1.372 | 1.488 | 1.478 | 0.000 |
| Histidine metabolism | 1.378 | 1.365 | 1.357 | 1.347 | 1.318 | 1.307 | 1.248 | 1.178 | 1.204 | 0.000 |
| Pantothenate and CoA biosynthesis | 1.324 | 1.337 | 1.332 | 1.314 | 1.290 | 1.297 | 1.291 | 1.252 | 1.256 | 0.000 |
| Carbon fixation in photosynthetic organisms | 1.322 | 1.311 | 1.331 | 1.338 | 1.290 | 1.260 | 1.253 | 1.236 | 1.197 | 0.000 |
| Porphyrin and chlorophyll metabolism | 1.421 | 1.377 | 1.445 | 1.481 | 1.270 | 0.989 | 1.001 | 1.130 | 0.993 | 0.000 |
| Lipid biosynthesis proteins | 1.211 | 1.198 | 1.161 | 1.158 | 1.229 | 1.260 | 1.281 | 1.285 | 1.298 | 0.000 |
| Terpenoid backbone biosynthesis | 1.142 | 1.165 | 1.126 | 1.138 | 1.166 | 1.204 | 1.185 | 1.180 | 1.222 | 0.016 |
| Glycerophospholipid metabolism | 1.084 | 1.073 | 1.045 | 1.064 | 1.115 | 1.135 | 1.162 | 1.182 | 1.200 | 0.000 |
| Pentose and glucuronate interconversions | 1.130 | 1.127 | 1.215 | 1.198 | 1.079 | 0.943 | 0.931 | 0.969 | 0.910 | 0.000 |
| Lipopolysaccharide biosynthesis proteins | 0.998 | 0.984 | 0.942 | 0.885 | 1.063 | 1.138 | 1.228 | 1.081 | 1.021 | 0.000 |
| Glyoxylate and dicarboxylate metabolism | 1.054 | 1.064 | 1.061 | 1.052 | 1.045 | 0.961 | 0.997 | 0.985 | 0.931 | 0.000 |
| Other glycan degradation | 1.121 | 1.152 | 1.252 | 1.206 | 0.998 | 0.972 | 0.886 | 0.735 | 0.748 | 0.000 |

* Only significantly changed functions (*p*.adjust < 0.05) with the relative abundance higher than 1% were shown.
